# ﻿*Staurantherafloribunda*, a new species of Gesneriaceae from Yunnan, China

**DOI:** 10.3897/phytokeys.232.108996

**Published:** 2023-09-07

**Authors:** Fan Su, Xiao-Wei Qin, Yun-Lei Wang, Ren-Fen Wang, Chao-Yun Hao, Ke Tan

**Affiliations:** 1 Spice and Beverage Research Institute, CATAS, Wanning 571533, Hainan, China Spice and Beverage Research Institute Wanning China; 2 Ministry of Agriculture Key Laboratory of Genetic Resources Utilization of Spice and Beverage Crops, Wanning 571533, Hainan, China Ministry of Agriculture Key Laboratory of Genetic Resources Utilization of Spice and Beverage Crops Wanning China; 3 Hainan Provincial Key Laboratory of Genetic Improvement and Quality Regulation for Tropical Spice and Beverage Crops, Wanning 571533, Hainan, China Hainan Provincial Key Laboratory of Genetic Improvement and Quality Regulation for Tropical Spice and Beverage Crops Wanning China; 4 Guangxi Key Laboratory of Plant Conservation and Restoration Ecology in Karst Terrain, Guangxi Institute of Botany, Guangxi Zhuang Autonomous Region and Chinese Academy of Sciences, Guilin 541006, Guangxi, China Guangxi Institute of Botany, Guangxi Zhuang Autonomous Region and Chinese Academy of Sciences Guilin China; 5 Gesneriad Committee of China Wild Plant Conservation Association, National Gesneriaceae Germplasm Resources Bank of GXIB, Gesneriad Conservation Center of China (GCCC), Guilin 541006, Guangxi, China Gesneriad Committee of China Wild Plant Conservation Association Guilin China

**Keywords:** China, Flora of Yunan, Gesneriaceae, new taxon, *Stauranthera*

## Abstract

*Staurantherafloribunda* F.Su, C.Y.Hao & K.Tan, a new species of Gesneriaceae from Yunnan, China, is described and illustrated here. It is morphologically similar to *S.grandifolia* Benth. in the shape of corolla, stigma, leaves and the number of stamens. However, it can be readily distinguished from the compared species by its dense cymes, leaf indumentum, lack of a corolla spur, calyx colour and stamen shape. The description of the new species, photographs, detailed descriptions, notes on etymology, distribution and habitat, as well as comparisons with morphologically similar species, are provided.

## ﻿Introduction

*Stauranthera* Benth. (1835) is a genus of perennial herbs in Gesneriaceae. Originally, this genus was established, based on the species *S.grandifolia* Benth. from the Malay Peninsula. The genus has previously been considered to comprise ca. 13 species distributed in Northeast India and South China throughout Malesia to New Guinea (Willis 1973; Burtt 1984). However, the taxonomic delimitation of the genus *Stauranthera* has been revised considerably over time, with *S.brandisii* C.B.Clarke being moved into *Rhynchotechum* Blume, *Staurantherajohannis-winkleri* Kraenzl. was synonymised with *S.argyrescens* Hallier f., *S.chiritiflora* Oliver and *S.tsiangiana* Hand-Mazz. were transferred to the new genus *Whytockia* W.W.Sm. in which *Staurantherachiritiflora* is the type of *Whytockia* (Smith 1919; Burtt 1984). Merrill (1923) synonymised both *Staurantheraecalcarata* R.Br. and *S.philippinensis* Elmer with *S.caerulea* (Blume) Merr. (Merrill 1923). Currently, the genus comprises ca. five species recognised by Möller et al. (2016), with two of them being recorded in China, namely *S.umbrosa* (Griffith) C.B.Clarke in Guangxi, Hainan and Yunnan and *S.grandifolia* Benth. in Yunnan and Hainan (Zhu et al. 1996; Qin and Xing 2006; Wei 2018; Wen et al. 2019; Huang and Liu 2023), respectively. Meanwhile, these two species were also documented in northern Vietnam, a region bordering the distribution area of *Stauranthera* in China (Wei et al. 2022). Additionally, *S.coerulea* (Blume) Merr., a Malesian species, was also introduced to northern Vietnam (Wei et al. 2022).

During a floristic expedition to Yunnan Province in 2022, the authors found an unknown species of Gesneriaceae near the Sino-Vietnamese border at Jinping County, China. Based on features like opposite, unequal leaves, small opposite bracts, a campanulate corolla tube with a basal spur, an indistinctly 2-lipped limb with a 2-lobed adaxial lip and 3-lobed abaxial lip, four included stamens and basifixed anthers connate into a shallow cone, this species appears to belong to the genus *Stauranthera*. However, detailed morphological comparisons to protologues and type specimens of all previously-described *Stauranthera* species revealed it does not match any known species. Thus, we confirmed that it as a new species, described and illustrated here as *S.floribunda* F.Su, C.Y.Hao & K.Tan. We provide a formal description of the new species and an updated key for the genus in China.

## ﻿Materials and methods

Morphological studies of the new species were based on the type specimens deposited in the Herbaria IBK and NPH and the living plants cultivated in the Spice and Beverage Research Institute, CATAS and Gesneriad Conservation Centre of China (GCCC). All available specimens of *Stauranthera* stored in the Herbaria AU, BM, E, G, HITBC, IBK, IBSC, K, KUN, PE and WU were examined from online specimen images via the Chinese Virtual Herbarium (CVH, https://www.cvh.ac.cn/index.php) and JSTOR (https://plants.jstor.org). Measurements of morphological characters were based on living plants whose photographs were taken with a Nikon D750 digital camera (Tokyo, Japan) and Dino-Lite digital microscope (Taiwan, China) and the morphological characters described using the terminology presented by Wang (1990) and Wang et al. (1998). Morphological comparison with close species was based on consultation with published literature. The conservation status evaluations of the new species *S.floribunda* were based on the International Union for Conservation of Nature Guidelines (IUCN 2022).

## ﻿Taxonomy

### ﻿ Stauranthera floribunda

Taxon classificationPlantaeLamialesGesneriaceae

F.Su, C.Y.Hao & K.Tan sp. nov.

E068B33D-D71B-58FA-9F28-0D8C155B54F7

urn:lsid:ipni.org:names:77326354-1

Figs 1 , 2

#### Diagnosis.

The new species resembles *Staurantheragrandifolia* in leaf blade shape and corolla, but can be easily distinguished from the latter in the flower number of cymes 2–4 bushes (vs. cymes 1–2 bushes), calyx colour white to slightly purple (vs. green), and bract 1, bracteole 1 auriculate connate (vs. bracts 2, linear opposite) and ovary 1–loculed (vs. 2–loculed) (Table 1).

**Table 1.** Morphological comparison of key characteristics in *S.floribunda* and *S.grandifolia*.

Characters	*S.floribunda*	*S.grandifolia*
Stem	6–28 cm tall, 5–12 mm in diam., puce, pulverulent	10–30 cm tall, 3–10 mm in diam., dark brown, pubescent
Leaf blade	leaves opposite, usually 3–5 pairs, with a normal leaf and a degenerated leaf at the internode, leaf blade strongly oblique, 14–28 cm long, 6–15 cm wide, adaxial surface glabrous, abaxial surface including leaf veins papillose-puberulent	leaves opposite, usually 2–5 pairs, with a normal leaf and a degenerated leaf at the internode, leaf blade strongly oblique, 12–27 cm long, 4–9 cm wide, abaxially pubescent or nearly glabrous, except leaf veins
Leaf vein	lateral veins 10–13 on the narrow side, 13–20 on the wide side	lateral veins 8–11 on the narrow side, 12–14 on the wide side
Petiole	4–6 cm long	1–3 cm long
Degenerated leaves	4–8 mm long	3–4 mm long
Bracts	bract 1, bracteole 1, auriculate connate	bracts 2, linear opposite
Cymes	2–4 bushes	1–2 bushes
Corolla	corolla purple or bluish-purple, throat yellowish; corolla tube ca. 5 mm long, orifice ca. 4 mm in diam.; no short spur	corolla blue-white, throat yellowish; corolla tube ca. 2.1 cm long, orifice ca. 10 mm in diam.; short spur at the base of the corolla
Calyx	white to slightly, purple	green
Stamens	4, adnate to ca. 5 mm above the corolla base, filaments linear, lilac, smooth, outside two ca. 4 mm long, anthers about 1.2 mm long, sides connected to each other.	4, adnate to ca. 3 mm above the corolla base, filaments linear white pilose, outside two ca. 3 mm long, anthers about 1 mm, sides connected to each other.
Pistil	ca. 9 mm long	ca. 5 mm long
Ovary	subuliform, ca. 5 mm long, ca. 4 mm in diam., 1-located, glandular puberulent about the position from the top to 1/3 of the ovary, the rest of ovary glabrous	ovoid. ca. 2 mm long, 2-loculed, glandular puberulent, covering the entire upper portion of the ovary
Style	linear, sparsely glandular-puberulent from the bottom to 2/3 of the style, the rest of style glabrous; stigma 1, covered by densely brownish puberulent hairs.	style sparsely white pilose, short; stigma large-capitate or bilamellate

#### Type.

China. Yunnan Province: Jinping Miao, Yao and Dai Autonomous County, Jinshuihe Town, Nawo, Rubber Forest, 22°37′42.30″N, 103°07′08.89″E, 316 m alt., 1 June 2023, *Fan Su 2023061* (Holotype: IBK! IBK00451428; Isotypes: NPH! NPH 001940, NPH 001941, NPH 001942).

#### Description.

Perennial Herbs, terrestrial, not rhizomatous, up to 35 cm high. ***Stem*** 6–28 cm tall, 5–12 mm in diam., puce, pulverulent. ***Leaves*** opposite, usually three pairs, occasionally five pairs, with a normal leaf and a degenerated leaf at the internode, leaf blade strongly oblique, oblong to elliptic obovate, 14–28 cm long, 6–15 cm wide at the middle, apex acuminate, base cuneate on narrow side, broadly cuneate to rounded on wider side, margin nearly entire, adaxially glabrous, abaxially including leaf veins papillose-puberulent, lateral veins 10–13 on the narrow side; 13–20 on the wider side; petiole 4–6 cm long, ca. 4 mm in diam., puberulous; degenerated leaves, sessile, adaxially green, abaxially greenish, pubescent, cordate to auricular-cordate to auricular-renifor, 2–4 mm long, 8–12 mm wide, margin entire, apex retuse or emarginate, leaf vein 3 pairs. ***Inflorescence*** dichotomous cyme, emerging from the axils of the large leaves, 3–9 cymes per plant, dense flowers, 10–20, rare 5 flowers per cyme, peduncles, pedicels, bracts, calyx densely white pubescent, sticky, peduncle up to 18 cm high; pedicels 1.5–2 cm long; bract 1, bracteole 1, green, auriculate connated; calyx broadly campanulate, ca. 2 cm in diam., white to slightly purple, 5-lobed, lobes deltoid, margin entire, longitudinally wrinkled between lobes. ***Corolla*** slightly obliquely campanulate, purple or bluish-purple, throat yellowish; corolla tube ca. 5 mm long, orifice ca. 4 mm in diam., upper lip ca. 8 mm long, 2-lobed, lobes suborbicular, ca. 4 mm in diam., lower lip slightly smaller than the upper lip. ***Stamens*** 4, adnate to ca. 5 mm above the corolla base, glabrous, filaments linear, lilac, smooth, inside two ca. 5 mm long, ca. 0.5 mm in diam., outside two ca. 4 mm long, ca. 0.3 mm in diam.; anthers triangular to cordate, ca. 1.2 mm long, sides connected to each other, dorsal septum slightly raised. ***Pistil*** ca. 9 mm; ***Ovary*** yellowish, subuliform, ca. 5 mm long, ca. 4 mm in diam., 1-located glandular puberulent about the position from the top to 1/3 of the ovary, the rest of ovary glabrous; ***Style*** ca. 3 mm long, linear, sparsely glandular-puberulent from the bottom to 2/3 of the style, the rest of style glabrous; stigma 1, covered densely brownish puberulent hairs. ***Capsule*** globose to oblatoid, glabrous, brownish-purple when young, brownish-black when mature.

**Figure 1.**
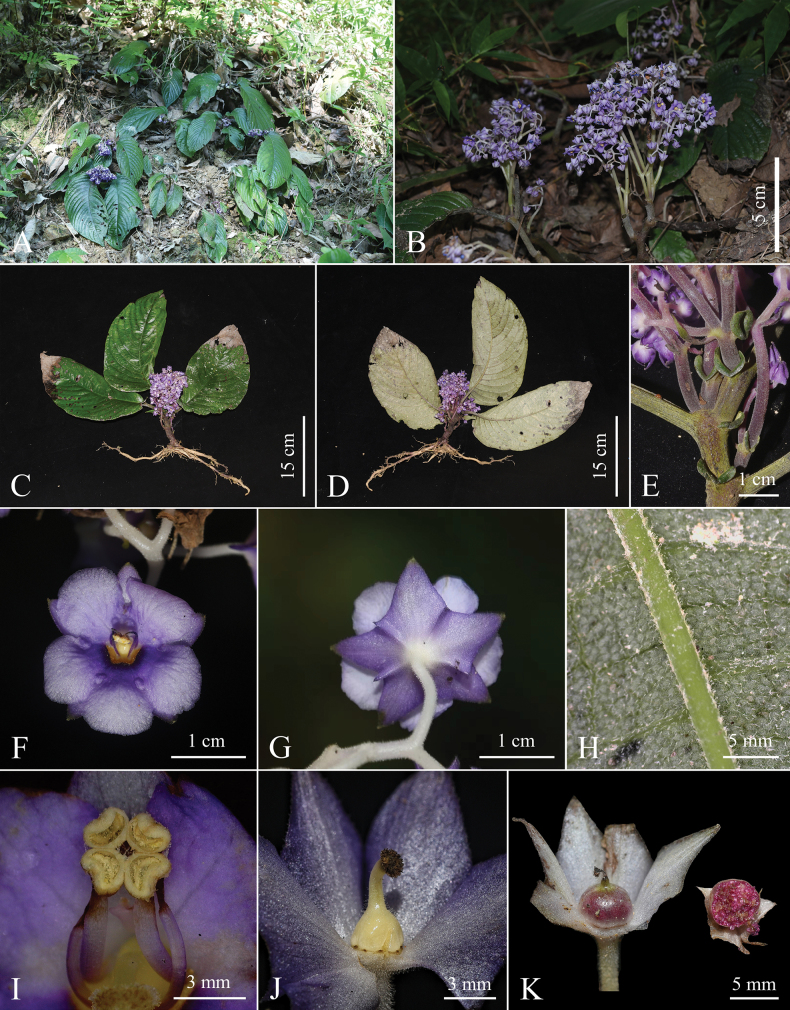
*Staurantherafloribunda* F.Su, C.Y.Hao & K.Tan, sp. nov. **A** habit **B** cymes **C, D** adaxial and abaxial views of the whole plant **E** degenerated leaves **F** front view of flower **G** back view of flower **H** detail of abaxial leaf blade **I** detail of stamens **J** detail of pistil **K** ovary and its cross-section view. Photographs by Fan Su.

#### Phenology.

Flowering in May and fruiting from June to July.

#### Etymology.

The specific epithet *floribunda*, which means “many-flowered”, refers the large numbers of the flowers per cyme and the whole plant of the new species. It is noticeably different from the flower numbers of all other known *Stauranthera* species.

**Figure 2.**
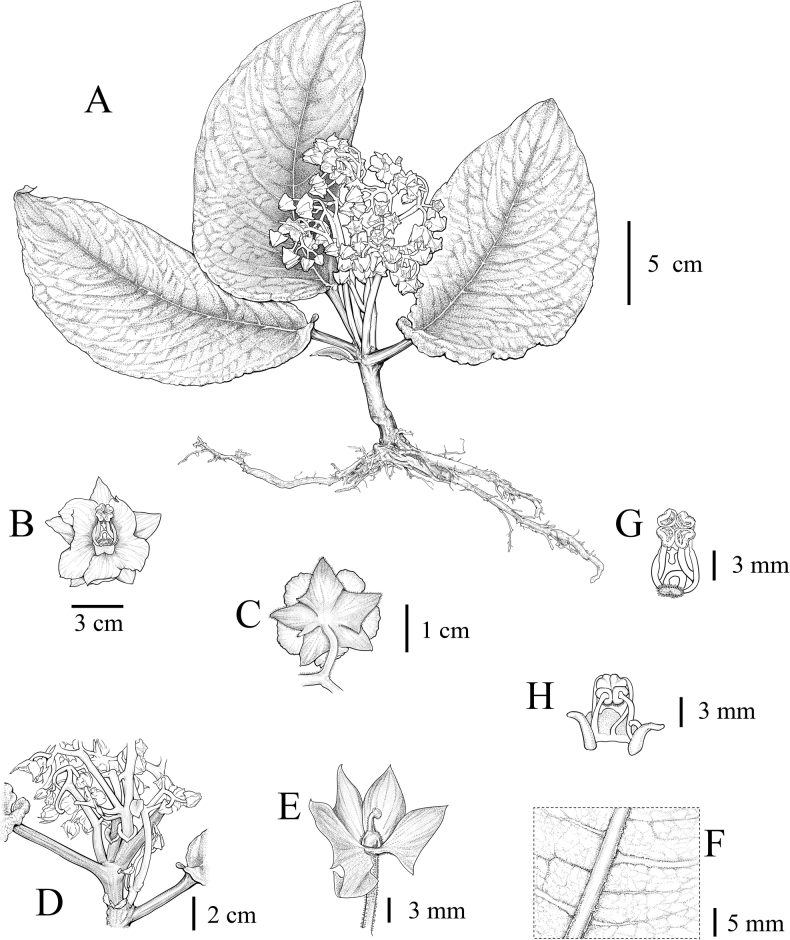
Line drawing of *Staurantherafloribunda* F.Su, C.Y.Hao & K.Tan **A** adaxial view of the whole plant **B** front view of flower **C** back view of flower **D** detail of degenerated leaves **E** pistil **F** hair of abaxial leaf blade **G** frontal view of stamens **H** dorsal side of stamen. Drawn by Di Hu.

#### Vernacular name.

Chinese: 多花十字苣苔 (duō huā shí zì jù taí). The name ‘duó huá’ means it has more flowers than other species in this genus and ‘shí zì jù taí’ is the Chinese name of *Stauranthera*.

#### Habitat and distribution.

Up to date, *Staurantherafloribunda* is only known in Nawo of Jinshuihe Town, Jinping County, at an elevation ca. 316 m, near Vietnam, south of Yunnan Province, China. The main companion species were *Alocasiaodora* (G.Lodd.) Spach, *Pellioniaradicans* (Siebold & Zucc.) Wedd., *Thunbergiagrandiflora* (Roxb. ex Rottler) Roxb. and *Oplismenuscompositus* (L.) P.Beauv.

#### Preliminary conservation status.

Since the only known population of *Staurantherafloribunda* is in the Rubber Forest of Jinshuihe Town, Jinping County, south Yunnan Province, we have not discovered the wild population expected from the above-mentioned location and information known about the population status and natural distribution range of the new species is very limited. Currently, less than 150 individuals have been found in the Rubber Forest. The new species of population, which grows close to a country road, is potentially threatened by human activities. Although no such habitat destruction is currently occurring, this population will likely be threatened in the foreseeable future under the influences of artificial factors, such as rubber cutting. Considering the known population is surrounded by rubber forests, it might survive under strong man-made intervention. Therefore, we suggest that the new species *S.floribunda* should be considered ‘**Critically Endangered**’ [**CR, B2a,b (iii,iv,v)**], facing a relatively high risk of extinction in the wild) according to current IUCN Red List Categories and Criteria (IUCN 2022).

### ﻿Key to the species of *Stauranthera* in China

**Table:** 

1	Bracts opposite, linear, corolla blue-white, upper lip ca. 7 mm long, 2-lobed, lower lip ca. 13 mm long, 3-lobed, short spur at the base of the corolla	***S.grandifolia***
–	1 bract, 1 bracteole, corolla white to purple or blue, upper lip ca. 8 mm long, 2-lobed, lower lip ca. 14 mm long, 3-lobed, no short spur at the base of the corolla	**2**
2	Cymes dense, calyx broadly campanulate, white to slight purple	***S.floribunda***
–	Cymes lax, calyx lobes broadly triangular, green	***S.umbrosa***

## Supplementary Material

XML Treatment for Stauranthera floribunda

## ﻿Additional information

### Author ORCIDs

Fan Su https://orcid.org/0000-0001-7223-6133

Xiao-Wei Qin https://orcid.org/0000-0002-4930-0632

Ren-Fen Wang https://orcid.org/0009-0009-7881-3080

Chao-Yun Hao https://orcid.org/0000-0002-7541-852X

Ke Tan https://orcid.org/0000-0002-9036-163X

### Data availability

All of the data that support the findings of this study are available in the main text.
